# Supplementary material for: Transcytosable Peptide-Paclitaxel Prodrug Nanoparticle for Targeted Treatment of Triple-Negative Breast Cancer
Source: Int J Mol Sci. 2023 Feb 28;24(5):4646. doi: 10.3390/ijms24054646 (PMC10003159; doi:10.3390/ijms24054646)
Supplement: Supplementary file 1 [file ijms-24-04646-s001.zip › ijms-2182495-supplementary.pdf]

Supporting Information

Transcytosable Peptide-Paclitaxel Prodrug Nanoparticle for Targeted Treatment of Triple-Negative Breast Cancer

Longkun Wang [1], Chunqian Zhao [1], Lu Lu [1], Honglei Jiang [1], Fengshan Wang [1,*] and Xinke Zhang [2,*]

[1] Key Laboratory of Chemical Biology (Ministry of Education), NMPA Key Laboratory for Quality Research and Evaluation of Carbohydrate-Based Medicine, Institute of Biochemical and Biotechnological Drug, School of Pharmaceutical Sciences, Cheeloo College of Medicine, Shandong University, Jinan 250012, China

[2] Key Laboratory of Chemical Biology (Ministry of Education), Department of Pharmacology, School of Pharmaceutical Sciences, Cheeloo College of Medicine, Shandong University, Jinan 250012, China

* Correspondence: fswang@sdu.edu.cn (F.W.), zhangxinke@sdu.edu.cn (X.Z.); Tel.: +86-0531-88382490 (F.W. & X.Z.)

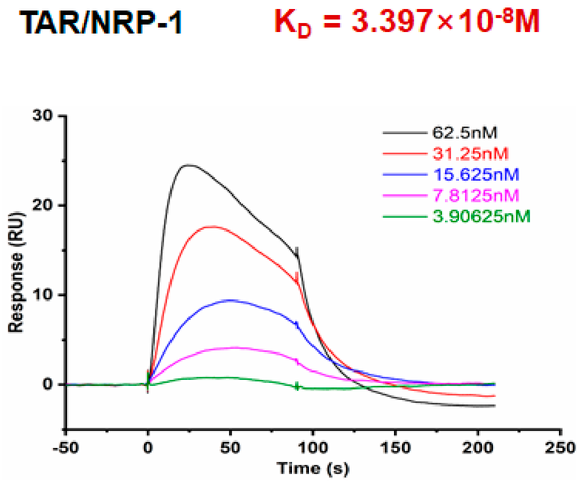

Figure S1. The binding curves for TAR peptide to the recombinant proteins NRP-1.

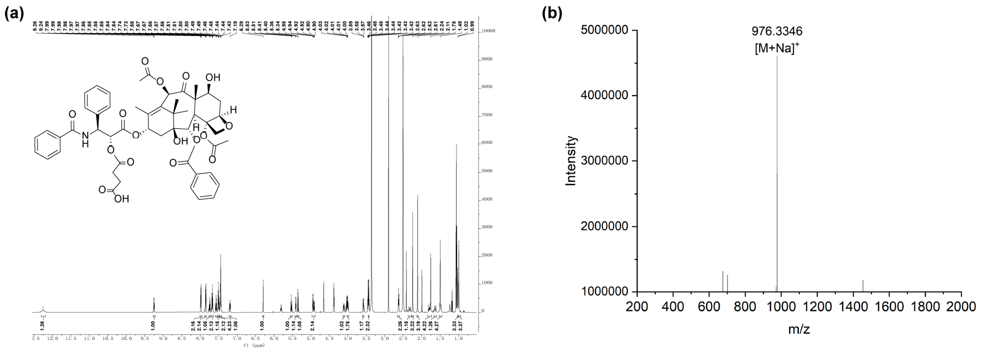

Figure S2. Characterization of PTX-SA. (**a**) The ^{1}H-NMR spectrum of PTX-SA (600MHz, DMSO-d$_6$). (**b**) The Q-TOF HRMS spectrum of PTX-SA.

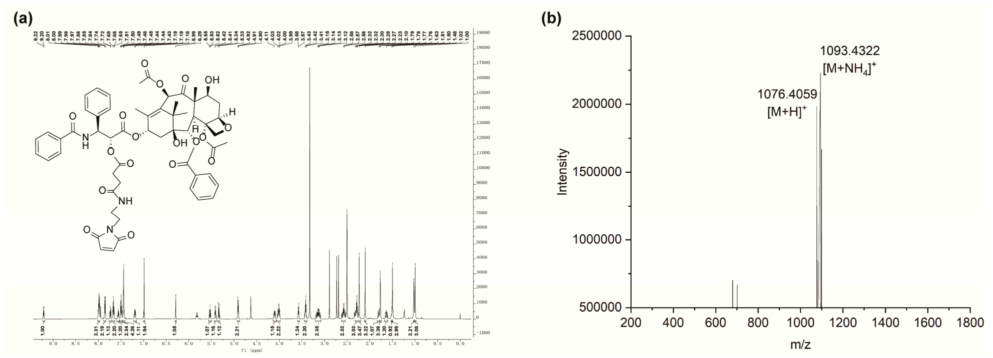

Figure S3. Characterization of PTX-SA-MAL. (**a**) The ^{1}H-NMR spectrum of PTX-SA-MAL (600MHz, DMSO-d$_6$). (**b**) The Q-TOF HRMS spectrum of PTX-SA-MAL.

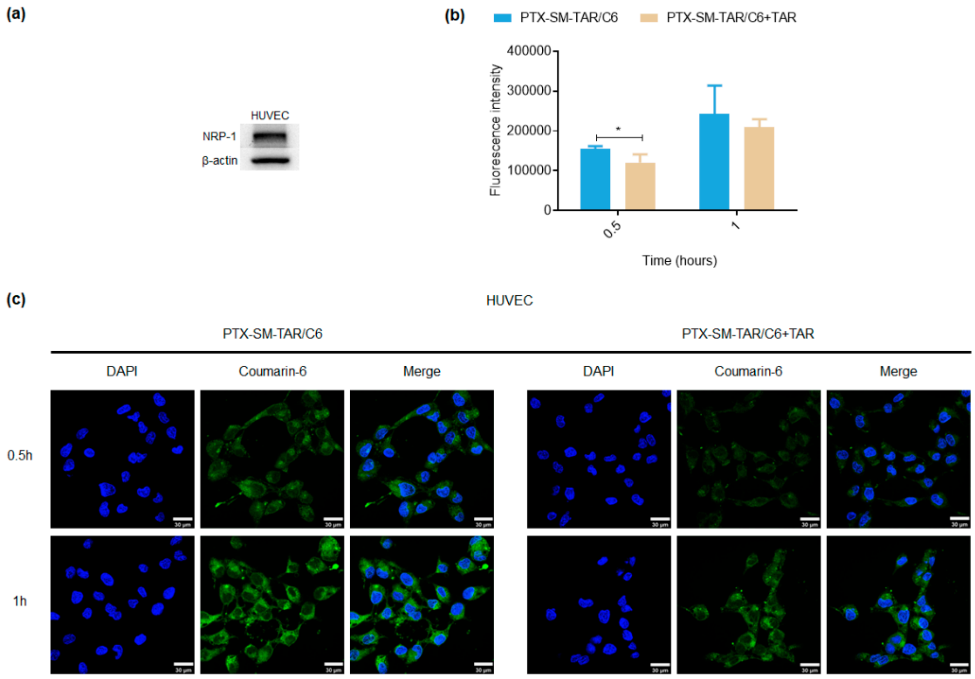

Figure S4. Cellular uptake of PTX-SM-TAR/C6 NPs in HUVEC cells. (**a**) Western blot analysis of NRP-1 expression in HUVEC cells. (**b**) Flow cytometry analysis of PTX-SM-TAR/C6 NPs and PTX-SM-TAR/C6 NPs plus TAR groups (mean ± SEM, $n = 3$, * indicates $p < 0.05$). (**c**) Confocal images of HUVEC cells. The cell nuclei were stained with DAPI (blue), and the PTX-SM-TAR NPs were dyed with coumarin-6 (green). The scale bar is 30 μm.

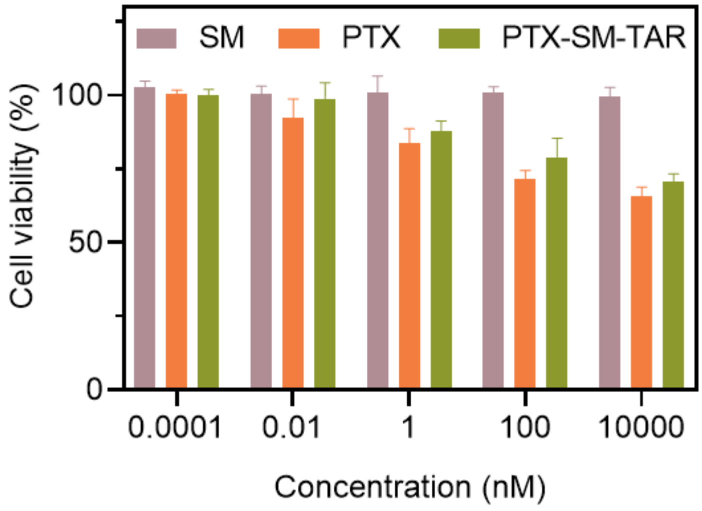

Figure S5. Cytotoxicity effect of SM, PTX, and PTX-SM-TAR NPs to EA.hy926 cells (mean ± SEM, $n = 3$).
